# Taxonomic revision of the freshwater mussel subtribe Cristariina (Bivalvia, Unionidae) in China: description of a new species and new synonyms

**DOI:** 10.3897/zookeys.1268.173464

**Published:** 2026-02-02

**Authors:** Hui Chen, Wei-Hua Qin, Wen-Hui Du, Hong-Quan Xiang, Yue-Ming He, Ge Guo, Ke-Lin Chu, Xiao-Ping Wu

**Affiliations:** 1 School of Life Sciences, Nanchang University, Nanchang, 330031, China College of Engineering and Technology, Tianjin Agricultural University Tianjin China; 2 Nanjing Institute of Environmental Sciences, Ministry of Ecology and Environment, Nanjing 210042, China School of Life Sciences, Nanchang University Nanchang China; 3 Department of Animal Science, Yuxi Agriculture Vocation-Technical College, Yuxi, 653106, China Nanjing Institute of Environmental Sciences, Ministry of Ecology and Environment Nanjing China; 4 College of Engineering and Technology, Tianjin Agricultural University, Tianjin, 300384, China Department of Animal Science, Yuxi Agriculture Vocation-Technical College Yuxi China; 5 Beijing Chaoyang RCF Dongba School, Beijing, 100018, China Beijing Chaoyang RCF Dongba School Beijing China

**Keywords:** Molluscs, phylogeny, Sichuan Basin, taxonomy, Unioninae

## Abstract

Although the subtribe Cristariina in China has been recently revised, taxonomic problems remain. This paper addresses these by describing a new species, *Cristaria liboi* Chen, Xiang, He & Guo, **sp. nov**., from Mianyang City, Sichuan Province, based on integrated morphological and phylogenetic analyses. Furthermore, we propose that *Cristaria beirensis* Y.-Y. Liu & W.-Z. Zhang, 1982 should be regarded as a junior synonym of *Cristaria plicata* (Leach, 1814), and that *Anemina arcaeformis* (Heude, 1877) is a junior synonym of *Anemina harlandi* (Baird & H. Adams, 1867), **comb. nov**.

## Introduction

Freshwater mussels of the family Unionidae perform crucial ecological functions (Vaughn and Hakenkamp 2001; Aldridge et al. 2007), yet they rank among the globally most imperilled groups of freshwater organisms (Lopes-Lima et al. 2020). The Chinese unionid fauna is remarkably diverse, with numerous species recorded in the Yangtze River Basin (Heude 1885; Simpson 1900; He and Zhuang 2013; Graf and Cummings 2021; Guo 2022; Chen et al. 2023; Dai et al. 2024a, 2024b; Chen et al. 2025). Although recent studies (Wu et al. 2025; Xiang et al. 2025) have offered a preliminary framework for the Chinese species of the subtribe Cristariini (Klishko et al. 2016; Wu et al. 2025), a comprehensive revision is still needed to resolve persistent issues. For instance, the relationship between *Anemina harlandi* (Baird & H. Adams, 1867), comb. nov. and *Anemina arcaeformis* (Heude, 1877), syn. nov. requires clarification. Furthermore, the taxonomic status of *Cristaria beirensis* Y.-Y. Liu & W.-Z. Zhang, 1982, syn. nov. is questionable (Wu et al. 2025), as records from Sichuan Province are geographically disjunct from its type locality in Hulunbuir City, Inner Mongolia. To address these issues, this study re-collected the aforementioned species and re-assessed their relationships through integrated morphological and phylogenetic analyses. Additionally, we describe a new species *Cristaria liboi* sp. nov.

## Materials and methods

### Specimen sampling and morphological observations

**Figure 1.**
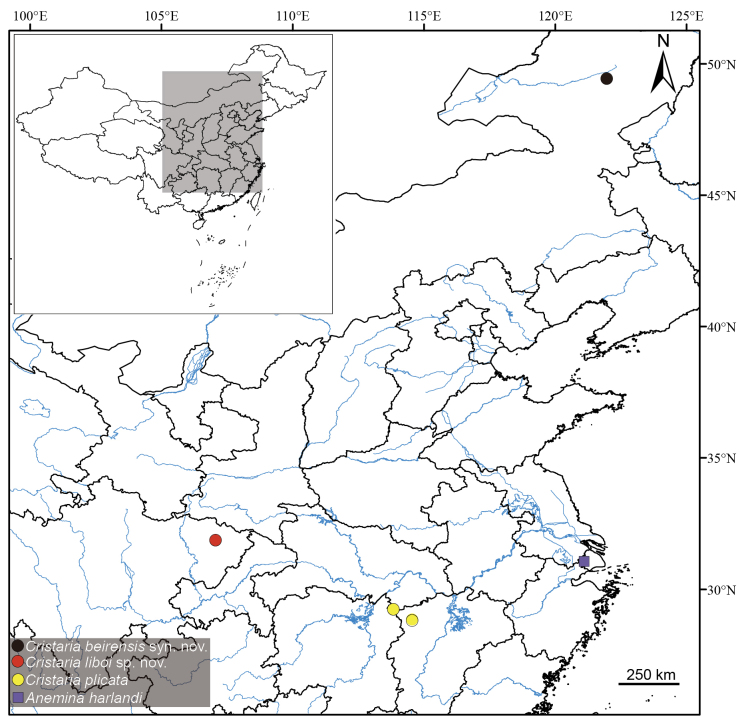
Sampling localities of specimens utilized in this study. The locality of *Cristaria beirensis*, which is treated herein as a junior synonym of *C. plicata* (syn. nov.), is distinctly marked. Symbols: black circle, *C. beirensis* (syn. nov. of *C. plicata*); red circle, *C. liboi* sp. nov.; yellow circle, *C. plicata*; purple square, *Anemina harlandi*.

**Figure 2.**
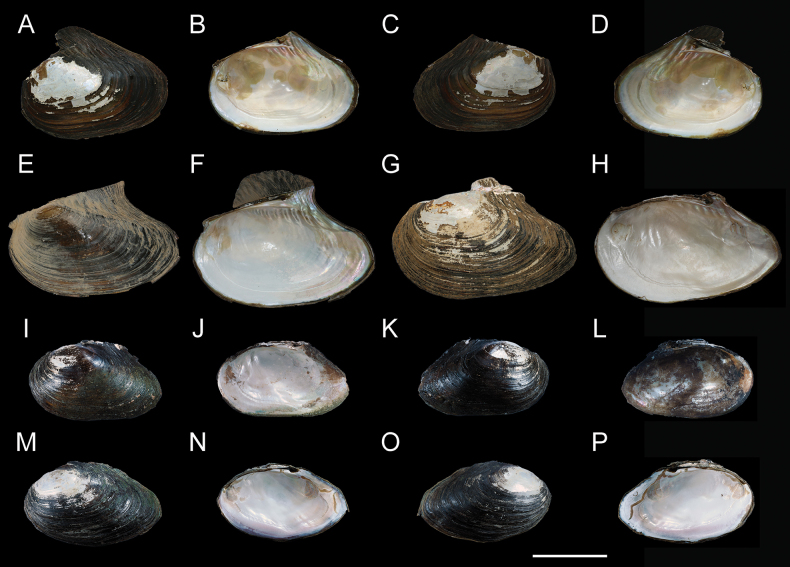
**A–H**. *Cristaria plicata* (Leach, 1814); **A–D**. *C. beirensis* syn. nov., NCUMB_CB251001, from Hexin Park, Hailar District, Hulunbuir City, Inner Mongolia, China; **E, F**. NCUMB_CB251002, from Yiyang City, Hunan Province, China; **G, H**. NCUMB_CB251003 from Changde City, Hunan Province; **I–P**. *C. liboi* Chen, Xiang, He & Guo, sp. nov. from Furong Creek, Mianyang City, Sichuan Province, China; **I–L**. Holotype, NCUMB_CL250901; **M–P**. Paratype: NCUMB_CL250902. Scale bar: 50 mm.

**Figure 3.**
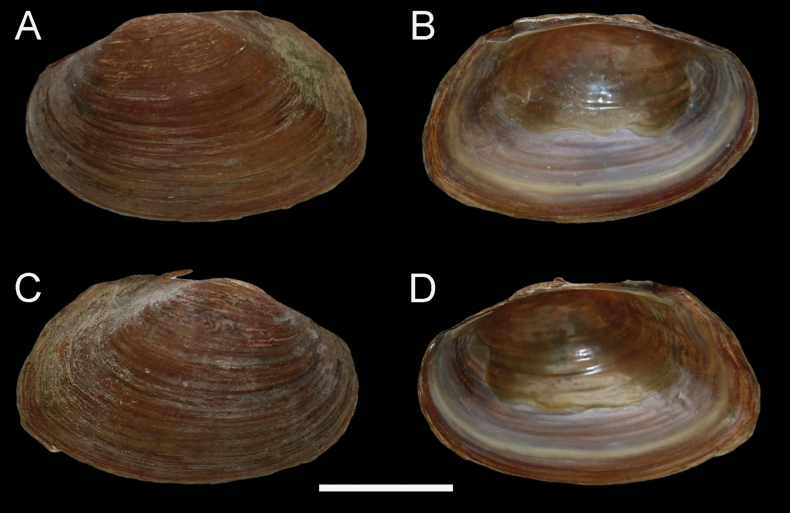
*Anemina harlandi* (Baird & H. Adams, 1867), NCUMB_AH251004, from Shanghai, China. Scale bar: 20 mm.

*Cristaria beirensis* Y.-Y. Liu & W.-Z. Zhang, 1982 were collected in Hexin Park (49.2083°N, 119.7722°E), Hailar District, Hulunbuir City, Inner Mongolia, China, in August 2025; *Cristaria plicata* (Leach, 1814) were collected in Yiyang City (28.5969°N, 112.3581°E) and Changde City (28.9823°N, 111.6459°E), Hunan Province, China, in August 2025; *Cristaria liboi* sp. nov. were collected in Furong Creek (31.6296°N, 104.8649°E), Mianyang City, Sichuan Province, China, in August 2025; *Anemina harlandi* (Baird & H. Adams, 1867), comb. nov. were collected in Chenshan Botanical Garden (31.08°N, 121.1849°E), Shanghai City, China, in August 2025 (Figs 1, 2, 3). The specimens were preserved in 95% ethanol, and deposited at the Museum of Biology, Nanchang University (**NCUMB**), China. Vernier callipers were used to measure shell length (**L**), width (**W**), and height (**H**).

### DNA extraction, PCR, sequencing, and phylogenetic analyses

Total genomic DNA was extracted on foot tissues using TIANamp Marine Animals DNA Kit (Tiangen Biotech) following the manufacturer’s protocol. Freshwater mussels exhibit doubly uniparental inheritance (DUI), a system in which highly differentiated maternal (F-type) and paternal (M-type) mitogenomes coexist. In males, the M-type genome is primarily localized in the germ line, whereas somatic tissues such as the foot are dominated by the F-type (Breton et al. 2007). As the majority of existing unionid COI barcode references are derived from somatic tissues (primarily the F-type), we targeted the F-type mitochondrial COI gene to ensure direct comparability. Amplification was performed using the primers LCO22me2 (GGTCAACAAAYCATAARGATATTGG) and HCO700dy2 (TCAGGGTGACCAAAAAAYCA) (Walker et al. 2007). Polymerase chain reaction (PCR) amplifications of COI were performed in a final 25 μL volume mixture containing 1 μL of template DNA, 1 μL of each pair of primers, 12.5 μL of Green Taq Mix (Vazyme, China), and 9.5 μL ddH2O. Thermal cycling began with one cycle at 98 °C for 10 s, followed by 35 cycles of denaturation at 94 °C for 1 min, 50 °C for 1 min, and 72 °C for 1 min, with a final extension step at 72 °C for 7 min. The PCR products were purified and sequenced using an ABI 3730XL analyzer. The newly obtained sequences were deposited in GenBank (Table 1). Sequences were aligned using MAFFT v. 7 based on the Q-INS-i method (Katoh et al. 2019). Pairwise distances between species were calculated using MEGA X (Kumar et al. 2018). The best substitution model was selected using the corrected Bayesian Information Criterion (BIC) in MODELFINDER v. 2.2.0 (Kalyaanamoorthy et al. 2017). Bayesian inference (BI) and maximum-likelihood (ML) analysis were performed using MrBayes v. 3.2.6 (Ronquist et al. 2012) and IQ-TREE v. 2.2 (Minh et al. 2020), respectively, with reference to the selected model of sequence evolution. For Bayesian analysis, two runs were performed simultaneously with four Markov chains starting from a random tree. Bayesian posterior probabilities (BPPs) of nodes were determined using Metropolis-coupled Markov chains (one cold chain) for 2 million generations, with sampling every 1000 generations. The first 25% of sampled trees were discarded as burn-in when the standard deviation of split frequencies of the two runs was less than 0.01; the remaining trees were then used to create a 50% majority-rule consensus tree and to estimate BPPs. Node support for maximum-likelihood analysis was determined using 1000 rapid bootstrap (BS) replicates.

**Table 1.** Details of the specimens and sequences used in the phylogenetic analysis. Sequences obtained for this study are marked by an asterisk.

Species	NCBI	Locality
*Cristaria bellua*	ON704642	Laos: Mekong basin, Nam Ngum River
	ON704643	Laos: Mekong basin, Nam Ngum River
*Cristaria clessini*	MT025813	Japan
	MT025814	Japan
*Cristaria liboi* sp. nov.	PQ387017	China: Sichuan
	PQ387018	China: Sichuan
	PX434362*	China: Sichuan, Mianyang, Furong Creek
	PX434363*	China: Sichuan, Mianyang, Furong Creek
*Cristaria radiata*	EU698909	
	EU698910	
*Cristaria truncata*	OP491287	Vietnam: Bang River
	OP491291	Vietnam: Bang River
*Cristaria plicata*	EU698907	
	EU698908	
	EU698912	
	EU698930	
*Cristaria plicata* syn. nov.	PX434364*	China: Inner Mongolia, Hulunbuir City
*Cristaria plicata* syn. nov.	PX434365*	China: Inner Mongolia, Hulunbuir City
	PX434366*	China: Hunan, Yiyang
	PX434367*	China: Hunan, Yiyang
	PX434368*	China: Hunan, Changde
*Pletholophus reinianus*	LC707540	Japan: Fukuoka, Munakata
	LC519027	Japan: Fukuoka, Munakata
*Pletholophus tenuis*	MT020601	Vietnam
	MT020602	Vietnam
*Pletholophus guangzhouensis*	PP945819	China: Guangdong, Ghuangzhou, Liuxi River
	PP945820	China: Guangdong, Ghuangzhou, Liuxi River
*Pletholophus honglinhensis*	MT020603	Japan
*Amuranodonta kijaensis*	MT020530	Russia
*Buldowskia suifunica*	MK574193	Russia: Primorsky Krai, Soldatskoe Lake
*Buldowskia flavotincta*	MT020538	South Korea
*Buldowskia kamiyai*	MT020525	Japan
*Buldowskia iwakawai*	MT020523	Japan
*Buldowskia shadini*	MK574200	Russia: Primorsky Krai, Melgunovka River
*Anemina harlandi*	MG462940	China: Hunan, Dongting Lake
	MG462942	China: Hunan, Dongting Lake
	PX434369*	China: Shanghai City, Chenshan Botanical Garden
	PX434370*	China: Shanghai City, Chenshan Botanical Garden
*Beringiana gosannensis*	MT020553	Japan
*Beringiana japonica*	MT020577	Japan
*Beringiana beringiana*	MK574222	USA: Alaska, Eugumen Lake
*Beringiana fukuharai*	OP028884	Japan: Gifu, Ena City
*Acudonta baitiaoensis*	PP891524	China: Sichuan
*Sinanodonta lucida*	KX822667	China
*Sinanodonta angula*	MG463057	China: Hunan, Anren County
*Sinanodonta tumens*	LC519023	Japan: Osaka, Yodo River
*Sinanodonta calipygos*	MT020623	Japan
*Sinanodonta schrenki*	MK574233	Russia: Primorsky Krai, Melgunovka River
*Sinanodonta woodiana*	MG463080	China: Jiangxi, Gan River
*Sinanodonta jourdyi*	KY561635	Vietnam
*Sinanodonta elliptica*	MG463065	China: Jiangxi, Gan River
*Sinanodonta pacifica*	MG591512	Malaysia: Padas basin
*Sinanodonta lauta*	MT020635	South Korea
*Cuneopsis celtiformis*	MG462964	China: Jiangxi, Gan River
*Mimo guoliangi*	PV091226	China: Yunnan, Luliang

## Results

**Figure 4.**
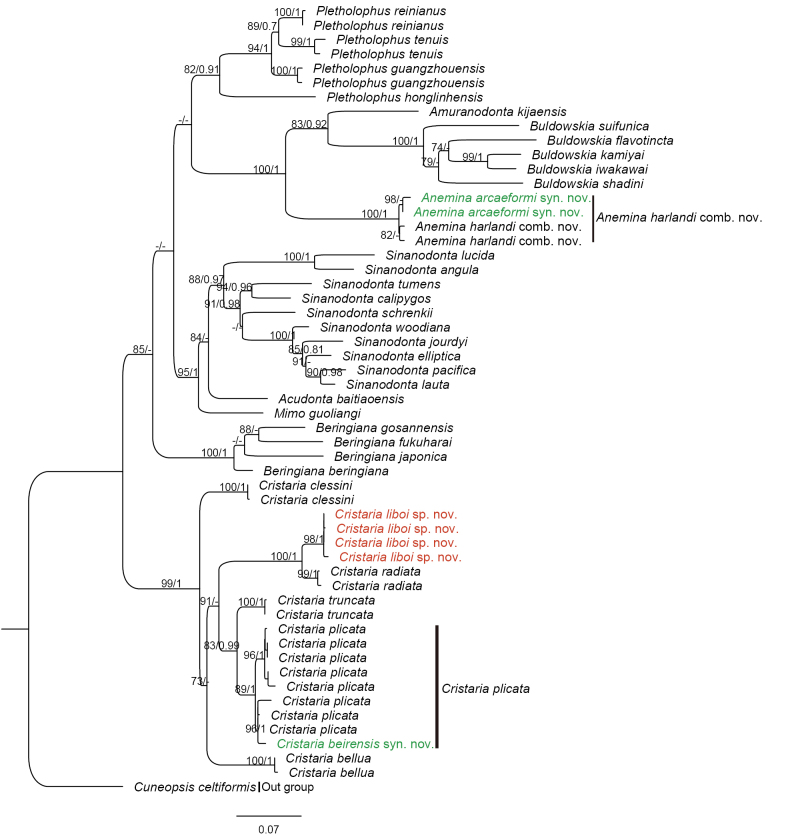
Maximum-likelihood (ML) phylogenetic tree based on cytochrome c oxidase subunit I (COI) gene sequences. Nodal support is indicated as ML bootstrap values (BS) and Bayesian posterior probabilities (PP). Only values ≥ 70% (BS) and ≥ 0.80 (PP) are shown. Species names are colored to highlight taxonomic findings: red for the new species (*Cristaria liboi* sp. nov.), and green for the junior synonyms (*C. beirensis* and *Anemina arcaeformis*).

The GTR+F+I+R4 model was selected as the best-fit model of nucleotide substitution by the AIC criterion. The phylogenetic analyses showed that the genus *Cristaria* formed a monophyletic clade with high support (BS = 99%, BPP = 1). The new species *Cristaria liboi* sp. nov. collected from Mianyang, Sichuan, China, together with *Cristaria radiata*, formed a strongly supported monophyletic clade (BS = 100%, BPP = 1) (Fig. 4). The uncorrected *p*-distances within the genus *Cristaria* based on COI sequences ranged from 3.3% to 12.7%. The smallest interspecific distance (3.3%) was observed between the new species *C. liboi* sp. nov. and its closest relative, *C. radiata*, while the largest distance (12.7%) was found between *C. liboi* and *C. bellua* (Table 2). These distances as well as the distinct morphological characteristics provide compelling evidence for its classification as a new species *C. liboi* sp. nov. in the genus *Cristaria*.

**Table 2.** Uncorrected *p*-distances (%) based on COI gene sequences among species of *Cristaria*.

	*C. clessini*	*C. liboi*	*C. radiata*	*C. truncata*	*C. plicata*	*C. bellua*
*Cristaria clessini*	0.2					
*Cristaria liboi*	12.3	0.1				
*Cristaria radiata*	11.8	3.3	0.1			
*Cristaria truncata*	7.9	11.3	11.1	0		
*Cristaria plicata*	8.2	10.6	9.8	4.9	1.1	
*Cristaria bellua*	8.7	12.7	12.1	8.7	8.8	0.3

### Systematics

#### Family Unionidae Rafinesque, 1820

**Subfamily Unioninae Rafinesque, 1820**

**Tribe Anodontini Rafinesque, 1820**

**Subtribe Cristariini Lopes-Lima, Bogan & Froufe, 2017**

##### Genus Cristaria

Taxon classificationAnimaliaUnionidaUnionidae

Schumacher, 1817

6FC71910-C201-5C99-822F-009482989FAC

###### Taxonomic note.

Based on morphological and molecular evidence presented in this study, *Cristaria beirensis* (Xiong & Liu, 1994) is herein treated as a junior synonym of *Cristaria plicata* (Leach, 1814), (syn. nov.). The following description and discussion of *C. plicata* encompass the characteristics of both nominal forms.

###### Type species.

*Cristaria plicata* (Leach, 1814).

##### Cristaria liboi

Taxon classificationAnimaliaUnionidaUnionidae

Chen, Xiang, He & Guo sp. nov.

B12DFFA4-544C-5E7B-BB36-7F5AA4BC3F01

https://zoobank.org/5220F1DA-BDEA-45BD-8938-E526A7D8E5F7

###### Material examined.

***Holotype***: • NCUMB_CL250901, shell length 185.21 mm, shell width 65.94 mm, shell height 111.25 mm (Fig. 2I–L), Furong Creek (31.6296°N, 104.8649°E), Mianyang City, Sichuan Province, China, in August 2025.

***Paratypes***: • 1 specimen, NCUMB_CL250902, shell length 185.13 mm, shell width 66.74 mm, shell height 113.85 mm (Fig. 2M–P), locality and habitat same as holotype.

###### Diagnosis.

Shell winged, large, oval, thick, solid; wing not obvious, posterior ridges high and obtuse. Umbo often eroded. Muscle scar obvious. Incurrent aperture without papillae arranged. Nacre lavender or white.

###### Description.

**Figure 5.**
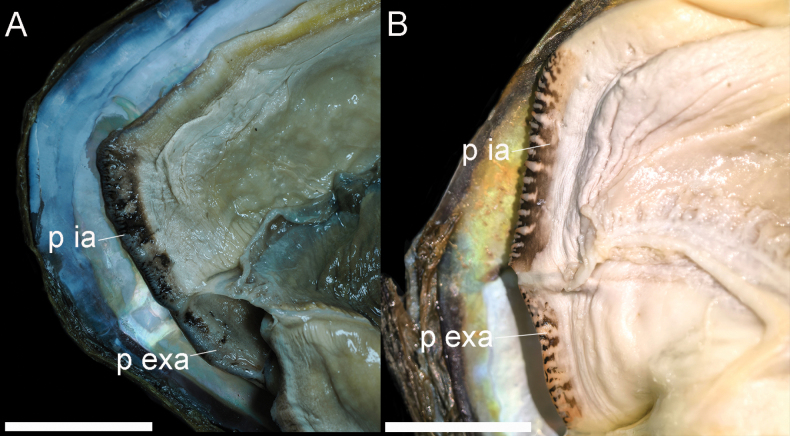
Internal anatomy of the mantle cavity of *Cristaria* species. **A**. *C. liboi* sp. nov.; **B**. *C. plicata*. Abbreviations: p ia = papillae of the incurrent aperture; p exa = papillae of the excurrent aperture. Scale bars: 30 mm.

Shell winged, large, oval, black, thick, solid; wing not obvious. Posterior ridges high and obtuse; ventral margin curved; posterior slope prominent; periostracum sculptured with thick and obvious concentric growth lines. Right and left valve with an almost completely degraded pseudocardinal teeth and a short lateral tooth. Hinge well developed. Umbo often eroded. Muscle scar obvious. Nacre lavender or white (Fig. 2I–P). Incurrent aperture without papillae arranged and excurrent aperture with obvious papillae arranged in one row; incurrent aperture papillae are more than twice the size of the excurrent aperture, and both have black pigmentation (Fig. 5A).

###### Etymology.

The name of this species is in honour of Mr Li Bo, a conchologist who collected the specimens.

###### Vernacular name.

李氏冠蚌 (li shi guan bang).

###### Habitat and distribution.

This species is currently only known to be distributed in the Furong Creek, Mianyang City, Sichuan Province (Fig. 1).

###### Remarks.

The species can be distinguished from other species in the genus by its characteristics, such as the incurrent aperture without arranged papillae and the wings being not obvious (Fig. 5A, B).

##### Cristaria plicata

Taxon classificationAnimaliaUnionidaUnionidae

(Leach, 1814)

446B3527-5FB5-55E1-8BC7-B349947FD58F

Anodon herculeus G.B. Sowerby II, 1867: pl. 3, fig. 7.
Anodonta (Dipsas) herculea von Middendorff, 1848: 302–304.
Anodonta cristata Blainville, 1825: 631.
Anodonta dipsas Blainville, 1825: 538, pl. 66, fig. 3.
Anodonta spatiosa Clessin, 1876: 173, pl. 57, fig. 2.
Craspedodonta smaragdina (Anton, 1838)—Clessin 1876: 93.
Cristaria beirensis Y.-Y. Liu & W.-Z. Zhang, 1982: 35. Syn. nov.
Cristaria (Cristaria) plicata (Leach, 1814)—Haas 1969: 387.
Cristaria herculea (Middendorff, 1847)—Vinarski & Kantor, 2016: 45.
Cristaria tuberculata Schumacher, 1817: 22.
Dianisotis chinensis Rafinesque, 1831: 7.
Dipsas bialatus (I. Lea, 1829)—Morle 1877: 266.
Dipsas occidentalis Heude, 1885: pl. 66, fig. 129.
Dipsas plicata Leach, 1814: 120, pl. 53.
Dipsas plicatus Leach, 1814: 120, pl. 53.
Symphynota bialata I. Lea, 1829: 445, pl. 14, fig. 24.
Symphynota magnifica I. Lea, 1834 *sensu* Mabille & le Mesle, 1866—Brandt 1974: 278.
Unio (Anodonta) smaragdinus Anton, 1838: 16.
Unio smaragdina Anton, 1838: 16.

###### Material examined.

• *Cristaria beirensis* syn. nov., NCUMB_CB251001, Hexin Park, Hailar District, Hulunbuir City, Inner Mongolia, China (49.2083°N, 119.7722°E) (Fig. 2A–D); • *C. plicata*, NCUMB_CB251002, collected in Yiyang City (28.5969°N, 112.3581°E) (Fig. 2E, F), NCUMB_CB251003, collected in Changde City (28.9823°N, 111.6459°E), Hunan Province, China (Fig. 2G, H).

###### Diagnosis.

Shell winged, large, oval, solid; wing obvious. Muscle scar obvious. Nacre lavender or white.

###### Habitat and distribution.

This species is widespread in China, Russia, and Japan where it lives in freshwater ponds, streams, reservoirs, and lakes, typically on soft substrates composed of sand and mud.

###### Remarks.

Our specimens of *C. beirensis* were collected from near its type locality. Both morphological comparison (Fig. 2A–H) and molecular evidence from the phylogenetic tree (Fig. 4) demonstrate a lack of significant differences. Therefore, we formally regard *Cristaria beirensis* as a junior synonym of *Cristaria plicata*.

##### Genus Anemina

Taxon classificationAnimaliaUnionidaUnionidae

F. Haas, 1969

FE4DF734-067D-537A-AC04-59006C0CF0D7

###### Type species.

*Anodon arcaeformis* Heude, 1877 accepted as *Anemina harlandi* (Baird & H. Adams, 1867), comb. nov.

*Anemina harlandi* (Baird & H. Adams, 1867), comb. nov.

*Anemina arcaeformis* (Heude, 1877)—Wu et al. 2025: 39. Syn. nov.

*Anemina euscaphys* (Heude, 1879)—He and Zhuang 2013: 31.

*Anemina fluminea* (Heude, 1877)—He and Zhuang 2013: 32.

*Anemina globosula* (Heude, 1878)—He and Zhuang 2013: 33.

*Anodon arcaeformis* Heude, 1877: pl. 19, fig. 40.

*Anodon euscaphys* Heude, 1879: pl. 35, fig. 68.

*Anodon fluminea* Heude, 1877: pl. 20, fig. 42.

*Anodon globosula* Heude, 1878: pl. 25, fig. 54.

*Anodonta harlandi* Baird & H. Adams, 1867: 492, pl. 26, fig. 3a.

*Anodon torrentis* Heude, 1878: pl. 29, fig. 61.

Anodonta (Haasiella) arcaeformis (Heude, 1877)—Korobkov 1954: 80.

Anodonta (Haasiella) euscaphys (Heude, 1879)—Korobkov 1954: 80.

*Anodonta arcaeformis* (Heude, 1877)—Lindholm 1925: 137.

###### Material examined.

• *Anemina harlandi* comb. nov., NCUMB_AH251004, 05, were collected in Chenshan Botanical Garden (31.0800°N, 121.1849°E), Shanghai City, China (Fig. 3).

###### Diagnosis.

Shell medium-sized, expanded, oval, thin, sub-glossy, opaque. Umbo inflated. Without pseudocardinal teeth.

###### Habitat and distribution.

This species is widespread in China where it lives in freshwater ponds, streams, reservoirs, and lakes.

###### Remarks.

Specimens of *Anemina harlandi* comb. nov. were collected near the type locality (Figs 1, 3) and correspond well to the illustrations provided in the original description. Morphological comparisons reveal no significant differences in shell shape between *Anemina harlandi* comb. nov. and *Anemina arcaeformis*. This conclusion is further supported by the phylogenetic analysis (Fig. 4). Therefore, *Anemina arcaeformis* syn. nov. is herein proposed as a junior synonym of *Anemina harlandi* comb. nov. Currently, this species represents the only extant member of the genus *Anemina* F. Haas, 1969.

## Discussion

This study involved sampling near the type locality of *Cristaria beirensis* (Fig. 2A–D), and through phylogenetic tree analysis (Fig. 4), it was confirmed that *C. beirensis* is a junior synonym of *Cristaria plicata*. Currently, there are four species of *Cristaria* recorded in China: *C. truncata* Dang, 198, *C. plicata*, *C. liboi* sp. nov., and *C. radiata* (Simpson, 1900). The species diversity of *Cristaria* in China is mainly concentrated in the Yangtze river basin, with *C. plicata* being most widespread. In northern China, *Cristaria* species are very limited, with only *C. plicata* present. Its shell morphology exhibits certain plasticity in different regions (Klishko et al. 2016). For *C. plicata* in different regions, there are slight variations in the incurrent and excurrent apertures. The taxonomic basis for recent records of *C. radiata* (e.g. Wu et al. 2025) is unreliable, as the source of the COI sequence (Jia et al. 2009) did not provide voucher imagery or a morphological identification to confirm the species assignment. Therefore, future investigations should involve collecting specimens from the type locality to determine the true identity of *C. radiata*.

A recent study proposed *Anemina arcaeformis* as the only species within the genus *Anemina*. However, the authors of that study overlooked the taxon historically known as *Anodonta harlandi*. Although *A. harlandi* had been treated as a junior synonym of *Sinanodonta woodiana* (I. Lea, 1834), its shell morphology is congruent with specimens identified as *A. arcaeformis* by Wu et al. (2025). To clarify this taxonomic confusion, we collected new topotypic specimens (from the type locality of *A. harlandi*, Figs 1, 3) matching the original description. Our molecular phylogenetic analyses (Fig. 4) conclusively demonstrate that *A. arcaeformis* is not a distinct species but is conspecific with *A. harlandi*. Therefore, we herein establish the revised classification: *Anemina harlandi* (Heude, 1877), comb. nov., with *A. arcaeformis* as its junior synonym (syn. nov.).

Based on the current research, species in the subtribe Cristariini that are endemic to China often have very narrow distributions, making them highly susceptible to human activities, leading to their potential endangerment. Some species may already be extinct (Xiang et al. 2025), so further systematic investigations of their distribution are necessary, along with the development of corresponding conservation measures.

## Supplementary Material

XML Treatment for Cristaria

XML Treatment for Cristaria liboi

XML Treatment for Cristaria plicata

XML Treatment for Anemina

## Additional information

### Use of AI

No use of AI was reported.

### Author ORCIDs

Hui Chen https://orcid.org/0009-0004-5222-3975

Hong-Quan Xiang https://orcid.org/0009-0005-6737-3092

Yue-Ming He https://orcid.org/0009-0002-9849-7977

Ge Guo https://orcid.org/0009-0000-4238-6798

Ke-Lin Chu https://orcid.org/0000-0001-5673-5514

### Data availability

The nucleotide sequence data generated and analyzed in this study have been deposited in the National Center for Biotechnology Information (NCBI) GenBank database. The accession numbers are provided in Table 1 of this article.
